# Galectin-3 is elevated in Müller glia in human glaucomatous eyes and ocular hypertensive rat eyes and associated with phagocytosing states

**DOI:** 10.1186/s40478-026-02388-7

**Published:** 2026-08-04

**Authors:** Anne Rombaut, Luozixian Wang, Emma Lardner, Raymond CB Wong, Charlotte Taul, Miriam Kolko, Gustav Stålhammar, Rune Brautaset, Filippo Locri, Pete A. Williams, James R. Tribble

**Affiliations:** 1 Department of Clinical Neuroscience, Division of Eye and Vision, St. Erik Eye Hospital, Karolinska Institutet, Stockholm, Sweden; 2 Centre for Eye Research Australia, Royal Victorian Eye and Ear Hospital, Melbourne, Australia; 3 Department of Surgery (Ophthalmology), The University of Melbourne, Melbourne, Australia; 4 Department of Drug Design and Pharmacology, University of Copenhagen, Copenhagen, Denmark; 5 Department of Ophthalmology, Copenhagen University Hospital, Glostrup, Denmark; 6 Department of Ophthalmology, Aalborg University Hospital, Aalborg, Denmark; 7 Wolfson Sensory, Pain and Regeneration Centre (SPaRC), School of Neuroscience, King’s College London, London, UK

**Keywords:** Retina, Glaucoma, Galectin-3, Astrocyte, Microglia, Müller glia, Neuroinflammation, Phagocytosis

## Abstract

Glaucoma is a leading cause of irreversible blindness worldwide, yet available treatments fail to prevent disease progression for all patients. It is characterized by a progressive dysfunction and loss of retinal ganglion cells. Neuroinflammation has been recognized as an underlying neurodegenerative mechanism of glaucoma in animal models and human post-mortem samples, and targeting neuroinflammation may provide additional means to neuroprotection. Galectin-3, a pro-inflammatory mediator encoded by the *LGALS3* gene in humans, holds promise as a treatable target as its pharmacological and genetic inhibition is neuroprotective in multiple models of experimental glaucoma. However, the role of Galectin-3 in glaucoma remains unclear, particularly whether its emergence is a consequence of degeneration, or occurs at earlier time points. To address these knowledge gaps, we labeled IBA1, GFAP, and Galectin-3 in retina sections at early glaucoma stages in the rat bead glaucoma model, and in human retina from glaucoma donors. In the rat, IBA1 volume, but not GFAP, increased at an early, pre-degenerative timepoint. Accompanying this, we identified a significant increase of Galectin-3/IBA1 colocalization compared to control at the same timepoint, supporting the upregulation of Galectin-3 in early inflammation, preceding retinal ganglion cell degeneration in experimental glaucoma. However, a significant increase in Galectin-3/GFAP colocalization compared to control at the same timepoint in the rat additionally associates Galectin-3 production with astrocytes and Müller glia. This Galectin-3 to Müller glia relationship was significantly pronounced in human glaucomatous retina, predominating over microglia co-labelling. We further demonstarted that human MIO-M1 Müller glia in vitro express Galectin-3, but this is not altered in response to glaucoma relevant stimuli (TNF-α or mild-metabolic stress from rotenone). Instead, Galectin-3 expression was altered in phagocytosing states from exposure to *E. coli* particles, brain synaptosomes, or apoptotic neuronal debris. These findings provide further insight into Galectin-3 and gross inflammatory responses in glaucoma pathology.

**Supplementary Information:**

The online version contains supplementary material available at https://doi.org/10.1186/s40478-026-02388-7.

## Introduction

Glaucoma is a common neurodegenerative disease characterized by the progressive loss of retinal ganglion cells, the neurons which transmit visual information to the brain. With at least 80 million patients currently estimated and 110 million expected by 2040 [39], glaucoma is a leading cause of irreversible blindness worldwide [17]. To date, lowering intraocular pressure (IOP), the major modifiable risk factor for glaucoma, remains the only available evidence-based treatment. However, IOP reduction does not cure the disease and fails to halt disease progression in a substantial proportion of treated patients [32]. It has been reported that up to 40% of glaucoma patients continue to lose vision in at least one eye despite treatment [36], underscoring the need to elucidate the mechanisms underlying glaucoma pathology and develop additional disease-modifying therapies.

Neuroinflammation is a common component of neurodegenerative diseases and has been recognized to significantly contribute to the progression of neurodegeneration in human patients and animal models [7]. In glaucoma there is evidence of neuroinflammation occurring across multiple animal models and human patients [26]. Whilst astrocytes and Müller glia (specialized radial glia in the retina) are mainly known for their retinal ganglion cell supportive functions, microglia are considered the resident macrophages of the retina. In experimental glaucoma, these glia have been demonstrated to drive and contribute to neuroinflammation in early disease pathology [49].

We previously identified an increase of Galectin-3 protein (also known as Mac-2), a member of the β-galactoside binding lectins, occurring at a degenerative time point in a rat bead model of glaucoma [9, 45]. Galectin-3 is associated with inflammatory responses in several systemic diseases and neurodegenerative diseases [8, 10, 20, 25, 38], and was demonstrated to be highly upregulated by disease associated microglia in two mouse models of glaucoma (mouse bead ocular hypertension and DBA/2J) at a neurodegenerative time point [15]. Apart from neuroinflammation, Galectin-3 is recognized as a marker for phagocytosis in both peripheral immune cells [31] and neurodegenerative contexts [3, 28]. Within these contexts Galectin-3 can also regulate phagocytosis in non-professional phagocytes, such as astrocytes. In experimental ischemia, Galectin-3 upregulation was identified in phagocytosing astrocytes [18] and in two mouse glaucoma models, Galectin-3 upregulation in a specific population of optic nerve head astrocytes was associated with a greater need for phagocytic clearance [19]. Genetic knockout (*Lgals3*^−/−^) or pharmacological inhibition via intravitreal injection of the small-molecule inhibitor 3,3-deoxy-3,3-bis-(4-[m-fluorophenyl]-1H1,2,3-triazol-1-yl)-thio-digalactoside (TD139) significantly ameliorated retinal ganglion cell loss in mouse [15] and rat [27] models of ocular hypertension. Additionally, the neuroprotective treatment with sitagliptin in a steroid-induced mouse glaucoma model resulted in significant downregulation of Galectin-3 in the optic nerve [2]. These findings indicate that Galectin-3 can play a pivotal role in the development and progression of glaucoma pathology. However, the neuroprotection after TD139 injections in glaucomatous rats was not accompanied by a change in gross neuroinflammation [27], suggesting that the role of Galectin-3 in glaucoma is yet to be fully resolved. Whether Galectin-3 upregulation occurs prior to neurodegeneration and whether this is a microglia specific response in the retina is unknown, as well as what effects Galectin-3 has on retinal glia in glaucoma pathology.

To address these knowledge gaps, we investigated the expression of glial markers at different timepoints of glaucoma development in the rat bead glaucoma model, where the time course of inflammation is yet to be characterized. We assessed which glia co-localize with Galectin-3, and whether this is consistent with human glaucomatous eyes. The data suggest that Galectin-3 upregulation occurs in astrocytes and Müller glia in addition to microglia, particularly in human glaucoma. By assessing Galectin-3 expression after inducing phagocytosis in cultured human Müller glia, we determined that phagocytosing Müller glia demonstrate dynamic Galectin-3 expression in a context dependent manner. Further investigation into the roles of Galectin-3 will be required if Galectin-3 targeting interventions are to be considered as future treatments for glaucoma.

## Methods

### Rat strain and husbandry

All experimental procedures were managed following the Association for Research for Vision and Ophthalmology Statement for the Use of Animals in Ophthalmic and Research. Individual study protocols were accepted by Stockholm’s Committee for Ethical Animal Research (10,389–2018 and 3909–2023). Adult, male Brown Norway rats (12–16 weeks old, weighing 300–375 g; SCANBUR, Karlslunde, Denmark) were housed in a regulated environment, (12 h light/12 h dark cycle) and fed with food and water ad libitum.

### Donor human eyes

Access to histopathology archive samples was fully covered through biobank #366 (St. Erik Eye Hospital, Stockholm, Sweden). For human retina explants, de-identified, post-mortem human eyes were obtained from the St. Erik eye hospital corneal eye bank as waste tissue following donation for corneal transplant. Sample IDs and subject information of histopathology and postmortem eyes used can be found in Supplementary data file 1. The study adhered to the tenets of the Declaration of Helsinki and the ethics protocols were approved by the Swedish Ethical Review Authority (2020–01525, amendments 2021–01036 and 2022–04511-02).

### Rat and human glaucoma eye histology

#### Immunofluorescent antibody labelling

Rat eyes from normotensive (NT) controls (n = 6), 3 days optical hypertension (OHT) (n = 6) and 7 days OHT (n = 7) were previously generated [47] using a microbead model as described before [46]. Briefly, OHT was induced bilaterally through injection of 4.5 μm diameter paramagnetic microbeads, with bilateral normotensive eyes as un-operated (naïve) normotensive controls. IOP was measured using a TonoLab rebound tonometer (Icare, Vantaa, Finland) in awake and unrestrained rats at day 0, day 3, and day 7 post OHT induction. Rats were euthanized at 3 or 7 days after OHT induction. The enucleated eyes were fixed in 3.7% formaldehyde in HBSS for 2 h, and prepared as paraffin blocks. We previously characterized a cohort of human eyes from glaucoma (n = 7) or ocular melanoma age and sex matched controls (n = 10) embedded in paraffin wax from a histopathology archive [30, 41]. New sections were cut for the present study on a wax microtome (Thermo Scientific Microm HM355S) at 3 μm thickness (anterior to dorsal plane), placed on glass slides, and baked at 60 °C for 1 h. Fluorescence immunohistochemistry was performed using a Bond-III fully automated IHC and in situ hybridization machine (Leica Biosystems Division of Leica Microsystems Inc., Bufalo Grove, IL). Sections were deparaffinized in Bond DeWax solution followed by rehydration through an ethanol gradient. Antigen retrieval was performed by immersing in 1 mM EDTA buffer (pH 8.9–9.1) for 30 min at 100 °C. The sections were then washed and incubated in primary antibody for 1 h at room temperature. Primary antibodies used were anti- ionized calcium-binding adaptor molecule 1 (IBA1) primary antibody (0.002 mg/mL, Abcam, cat. AB178846), anti- Glial fibrillary acidic protein (GFAP) primary antibody (1:500, Novus Biologicals, cat. NBP1-05198), anti Galectin-3 primary antibody (for human sections: 0.01 mg/ml, Invitrogen cat. 14–5301-85; for rat sections: 0.001 mg/ml, Biotechne R&D systems, cat. AF1197-SP). Slides were washed again and incubated in secondary antibodies (Cyanine-based Fluorescent dye 488 donkey anti-rabbit (0.004 mg/ml, Sigma-Aldrich, cat. SAB4600036), Alexa Fluor 647 donkey anti-chicken (0.004 mg/ml, Invitrogen, cat. A78952), and either Alexa Fluor 568 donkey anti-rat (0.004 mg/ml, Invitrogen, cat. A78946) for human sections or Alexa Fluor 555 donkey anti-goat (0.004 mg/ml, Invitrogen, cat. A21432) for rat sections for 15 min at room temperature followed by color development for 15 min. Sections were mounted from water with Fluorescence Mounting Medium (Agilent, cat. S302380-2) and covered with a coverslip.

#### Confocal microscopy, marker volume reconstruction, and co-localization

All images of the rat and human sections were acquired using a Zeiss LSM800 confocal microscope, using an objective of 20 × magnification and numerical aperture 0.8. The images captured were 319.45 × 319.45 μm. Imaging parameters were kept constant within species, within antibody conditions. For rat retina sections, images were captured at ± 1000 µm and ± 2500 µm from the optic nerve. For human retina sections, images were captured at ± 2000 µm, ± 4000 µm, and ± 6000 µm from the optic nerve. For control human eyes, only the retina opposite the ocular melanoma tumor was considered, where tumor effects on inflammation are limited [30]. The Imaris Surface tool was used to reconstruct the volume of the GFAP, IBA1 and Galectin-3 signal for both rat and human eyes. Only the signal in the inner retina was considered. For plotting, resulting volumes were normalized to the volume of the inner retina, and averaged per eye, per position of the image (same positions superior and inferior of the optic nerve were taken together). Colocalization was measured by constructing a colocalization channel in Imaris and extracting the percentages of signal colocalized above threshold.

### RNA-seq analysis of human retina

To assess Galectin-3 expression in normal human retina we accessed a NucSeq dataset [21, 22] through NCBI GEO (GSE135133). Data were imported into Seurat v4 for analysis. Using metadata provided by the authors we retained 100,055 high quality nuclei following QC filtering. Normalization was performed using the *NormalizeData()* function. Features with high cell–cell variation were identified using the *FindVariableFeatures()* function using the VST method, using default setting to return 2000 features. And linear transformation was applied to the dataset using *ScaleData()* function. Principal components were computed using the variable features and UMAP dimensional reduction was performed with the first 20 dimensions. Clustering and cell annotation ID provided by the authors were used to identify the major cell types in the retina, and unidentified subpopulations were removed from further analysis. Visualization of gene expression was generated using the *Dotplot()*, function in the Seurat package. We also assessed Galectin-3 expression in the Human Retina Cell Atlas v1.0 single-nucleus RNA-sequencing dataset obtained from the Human Cell Atlas data portal [12, 13]). The processed AnnData (h5ad) object, comprising 3.17 million nuclei from 122 donor retina samples, was analyzed using Python. Nuclei with available donor age metadata were retained. Cell clustering and annotations provided by the original study were used to identify major retinal cell classes. For Müller glia–specific analyses, nuclei annotated as Müller glia based on the original study annotations were subsetted from the full dataset, resulting in 216,093 Müller glia nuclei. Donors were grouped into three age categories (0–33, 34–66, and ≥ 67 years) and Müller glia were subsequently analyzed across donor age and age groups. Gene expression values were extracted directly from the expression matrix, and group-level statistics including mean expression and percentage of expressing nuclei were calculated. Gene expression distributions were visualized using custom Python scripts to generate dot plots.

### Human retinal explants, antibody labeling and imaging

Retinas were dissected free from post-mortem human eye cups and the vitreous was removed. Retinal punches were taken with a sterile disposable biopsy punch (2 mm diameter, MILTEX cat. 400–4420910) in an arc 3 mm nasally from the optic nerve head. Immediately after the axotomy, D0 punches were either fixed in 3.7% formaldehyde (≥ 36.0%, Sigma cat. 47,608-250ML-F) in HBSS for 1 h for immunolabeling of anti-RNA-binding protein with multiple splicing (RBPMS), or proteins were extracted for Western blot. D7 punches were placed ganglion cell layer up on culture inserts (Millicell 0.4 µm pore, Merck, cat. PICM01250) and cultured at 37 °C, 5% CO_2_ for 7 days in Neurobasal-A (Gibco, cat. 10,888–022) medium containing 2 mM GlutaMAX (Gibco, cat. 35,050–061), 2% B27 (Gibco, cat. 17,504–001), 1% N2 (Gibco, cat. 17,502–048), and 1% penicillin/streptomycin (Gibco, cat. 10,378–016). Half of the medium was changed on alternating days. At day 7, punches were removed from culture and either fixed in 3.7% formaldehyde for 1 h or proteins were extracted for Western blot.

For immunolabeling of RBPMS in the D0 and D7 retina punches, following fixation the punches were permeabilized with 0.5% Triton X (VWR Chemicals, cat. 28,817.295) in 1 × Phosphate-Buffered Saline (PBS) for 1 h at room temperature, incubated with 2% Bovine Serum Albumin (BSA, Sigma cat. A-4161) in PBS for 1 h at room temperature and incubated with anti-RBPMS primary antibody (Novus, cat. NBP2-80,389) overnight at 4 °C. Punches were washed 5 times in PBS for 5 min and secondary antibody Alexa Fluor 568 goat anti-guinea pig (0.004 mg/mL, Invitrogen, cat. A11075) was applied for 4 h at room temperature. After the incubation, punchess were washed again five times for 5 min in PBS and Hoechst nuclear stain (0.005 mg/mL, Invitrogen cat. H1399) was applied for 10 min. Images of RBPMS immunolabeling in human retina punches were acquired using a Leica DMi8 microscope with a CoolLED pE-300 white LED-based light source and a Leica DFC7000 T fluorescence color camera (all Leica). 2 images were acquired per punch, to cover roughly the whole punch area but excluding edges. The images were captured using an objective of 20 × magnification and numerical aperture 0.4, resulting in 665.6 × 665.6 µm images. Imaging parameters were kept constant within antibody conditions. The RBPMS + cells were counted using the Cell Counter plugin in ImageJ (v. 2.16.0/1.54p), and averaged per donor for calculation of retinal ganglion cell degeneration.

### SY5Y cell culture

SH-SY5Y, a human neuroblastoma cell line, was grown in FBS medium (DMEM with 1 g/L glucose and GlutaMAX (Gibco, cat. 31,966–021), supplemented with 1% penicillin–streptomycin (Gibco, cat. 10,378–016) and 10% fetal bovine serum (FBS: Gibco, cat. A5256801). SH-SY5Y cells were maintained at 37 °C, 5% CO_2_ in a humidified incubator until the cells reached 80% confluency. The cells were detached for seeding at a density of 240,000 cells/well in 6-well plates by treatment with Trypsin–EDTA 0.25% (Gibco, cat. 25,200–072).

SH-SY5Y cells were differentiated to a more neuronal phenotype by replacing the 10% FBS medium with DMEM (Gibco, cat. 31,966–021) medium containing 2.5% FBS, 1% penicillin–streptomycin (Gibco, cat. 10,378–016) and 10 µM retinoic acid (Sigma, cat. R2625) (M1) 24 h after seeding. The M1 medium was replaced by M2 medium on day 6 after the start of differentiation, consisting of Neurobasal-A without L-glutamine (Gibco, cat. 10,888–022) supplemented with 2% B27 (Gibco, cat. 17,504–001), 2 mM GlutaMAX (Gibco, cat. 35,050–061) and 1% penicillin–streptomycin (Gibco, cat. 10,378–016). 11 days after the start of SH-SY5Y differentiation, the cells were prepared for induction of MIO-M1 phagocytosis (see below).

### Müller glia culture

MIO-M1, a human Müller glial cell line, was grown in FBS medium (DMEM with 1 g/l glucose and GlutaMAX (Gibco, cat. 32,430–027), supplemented with 1% penicillin–streptomycin (Gibco, cat. 10,378–016) and 10% fetal bovine serum (FBS: Gibco, cat. A5256801). MIO-M1 cells were maintained at 37 °C, 5% CO_2_ in a humidified incubator until the cells reached 80% confluency. The cells were detached for seeding in cell plates by treatment with Trypsin–EDTA 0.25% 1x (Gibco, cat. 25,200–072).

#### Müller glia antibody labelling and imaging

For the immunofluorescent labeling of Galectin-3 in MIO-M1 Müller glia, cells were grown at a density of 50 000 cells/well (n = 3 for primary antibody positive, n = 3 for primary antibody negative), and fixed for 15 min in 3.7% formaldehyde in HBSS (≥ 36.0%, Sigma cat. 47,608-250ML-F) 20 h after seeding. The cells were permeabilized with 0.5% Triton X (VWR Chemicals, cat. 28,817.295) in 1 × Phosphate-Buffered Saline (PBS) for 15 min at room temperature, incubated with 2% Bovine Serum Albumin (BSA, Sigma cat. A-4161) in HBSS for 30 min at room temperature and incubated with anti-Galectin-3 primary antibody (0.01 mg/ml, Invitrogen, cat. 14–5301-85) overnight at 4 °C. Cells were washed 5 times in PBS for 5 min and secondary antibody Alexa Fluor 488 goat anti-rat (0.004 mg/mL, Invitrogen, cat. A11006) was applied for 2 h at room temperature. After the incubation, cells were washed again five times for 5 min in PBS and Hoechst nuclear stain (0.005 mg/mL, Invitrogen cat. H1399) was applied for 10 min. The cells were imaged in clean PBS in the cell plate. Images of Galectin-3 immunolabeling in MIO-M1 Müller glia were acquired using a Leica DMi8 microscope with a CoolLED pE-300 white LED-based light source and a Leica DFC7000 T fluorescence color camera (all Leica). 4 images were acquired per well, at an equal distance to the center. The images were captured using an objective of 20 × magnification and numerical aperture 0.4, resulting in 665.6 × 665.6 µm images. Imaging parameters were kept constant within antibody conditions. The intensity of the Galectin-3 signal was calculated using the Measuring tool (mean gray value) in ImageJ after thresholding, and averaged per well for Galectin-3 labeling before plotting.

#### Galectin-3 enzyme-linked immunosorbent assay (ELISA)

Galectin-3 secretion by MIO-M1 cells in the medium was assessed with a commercially available human Galectin-3 ELISA kit (Abcam, cat. Ab269555). Cells were grown for 72 h at a density of 45 000 cells/well, and 500 µl undiluted medium was collected per well, snap frozen and stored at -20 °C until further processing. Briefly, samples were thawed, spun in a centrifuge at 2000 × g, and supernatant was collected. Reagents and Galectin-3 standards were made according to kit instructions. 50 µl undiluted sample or standard and 50 µl antibody cocktail was added to appropriate wells, and incubated for 1 h at room temperature on a plate shaker (400 rpm). Next, each well was washed 3 times for at least 10 s with 350 µl 1 × Wash Buffer PT. After the washes 100 µl of TMB Development Solution was added per well, and incubated for 10 min in the dark on a plate shaker (400 rpm), after which the development was stopped with 100 µl of Stop Solution per well and the optical density of the plate was read at 450 nm on a plate reader (infinite® 200, Tecan). The optical density data of a Galectin-3 standard curve was used to extrapolate the Galectin-3 concentrations in pg/ml of the cell medium samples.

#### Exposure to glaucoma-relevant stimuli and cytotoxicity assessment

MIO-M1 cells were treated with TNF-α (R&D Systems, cat. 210 TA/CF) at 10 or 50 ng/ml in 1% FBS medium 24 h after seeding. 1% FBS medium without TNF-α was used as control. Cells were incubated at 37 °C, 5% CO_2_ and proteins were extracted for Western blot 24 h after treatment. MIO-M1 cells were treated with rotenone (Merck, cat. R8875) at 0.1, 1, 10, 100 or 1 µM in 1% FBS medium, maintaining a constant DMSO concentration of 0.25%, 24 h after seeding. 1% FBS medium containing no rotenone, but 0.25% DMSO was used as control. Cells were incubated at 37 °C, 5% CO_2_ and proteins were extracted for Western blot 24 h after treatment.

For cell death assessment, cells were fixed in 3.7% PFA and Hoechst nuclear stain (0.005 mg/mL, Invitrogen cat. H1399) was applied for 10 min. Whole well tile-scans of the Hoechst signal were acquired using a Leica DMi8 microscope with a CoolLED pE-300 white LED-based light source and a Leica DFC7000 T fluorescence color camera (all Leica). The images were captured using an objective of 5 × magnification and numerical aperture 0.12, resulting in 17,097.6171 × 17,134.0171 µm merged tile-scans. Imaging parameters were kept constant. Hoechst + cells per well were counted using the Particle Analysis tool in ImageJ, and statistically compared and plotted per condition.

#### Phagocytosis assays

All phagocytosis assay were executed by seeding the MIO-M1 cells at a density of 50,000 cells / well in 24 well plates.

For induction of MIO-M1 phagocytosis with pHrodo particles, pHrodo™ Red E. coli BioParticles™ (Invitrogen, cat. P35361) were reconstituted in HBSS and probe sonicated 3 times for 10 s at 40 Hz amplitude prior to use. 24 h after seeding, the medium was replaced with 1% FBS medium containing either 100 µg/ml pHrodo particles or no particles as a control. Cells were incubated at 37 °C, 5% CO_2_, and taken out to room temperature for short periods for automatic plate imaging. Images were acquired at 1, 2.5, 3, 4, and 24 h after treatment on a Leica DMi8 microscope as above (4 images per well, at an equal distance to the center). The intensity of the pHrodo signal was calculated using the Measuring tool (mean gray value) in ImageJ after thresholding and averaged per condition. 24 h after treatment, proteins were extracted for Western blot.

For induction of MIO-M1 phagocytosis with mouse cortex synaptosomes, fresh mouse cortex was isolated in Syn-PER™ Synaptic Protein Extraction Reagent (10 ml per gram of tissue, Thermo Scientific, cat. 87,793), homogenized with 10 strokes in a Dounce homogenizer followed by 10 min centrifugation at 1200 G and 4 °C. The supernatant was collected and centrifuged at 15,000 G and 4 °C for 20 min. The resulting synaptosome pellet was resuspended in 1% FBS medium and used to replace the MIO-M1 medium 24 h after seeding. A whole mouse cortex was used to treat 24 MIO-M1 wells, 1% FBS medium without synaptosomes was used as control. Cells were incubated at 37 °C, 5% CO_2_ and proteins were extracted for Western blot 24 h after treatment.

For induction of MIO-M1 phagocytosis with SH-SY5Y neurons, SH-SY5Y neurons were differentiated for 10 days (see above). On day 11, the cell medium was replaced by M2 medium containing 1 µM rotenone. 24 h after rotenone treatment, SH-SY5Y cells were scraped out of the well in ice cold DPBS without calcium or magnesium and centrifuged for 5 min at 5000 G to pellet. The supernatant was discarded, and the pellet resuspended DPBS. This was repeated to reach a total of 3 washes. The pellet was homogenized in DBPS using 10 strokes in a Dounce homogenizer, after which the homogenate was incubated for 30 min on ice. The homogenate was then centrifuged for 5 min at 5000 G, and the pellet was resuspended in 1% FBS medium for MIO-M1 treatment 24 h after seeding. All SH-SY5Y neurons present in a 6 well plate after 10 days of differentiation were used to treat 10 MIO-M1 wells, 1% FBS medium without SH-SY5Y neurons was used as control. Cells were incubated at 37 °C, 5% CO_2_ and proteins were extracted for Western blot 24 h after treatment.

To inhibit phagocytosis, cells were treated with cytochalasin D (1 µM, Sigma, cat. C2618) for 1 h before exposure, and subsequently for 4 h during exposure to synaptosomes or SHY5Y fragments. When cytochalasin D treatments were included, all treatment media were supplemented with 0.25% DMSO. Cells were washed with DPBS following the 4 h exposure and fresh DMEM with 1% FBS was added. Cells were harvested 20 h later.

#### Galectin-3 Western blot quantification

Cells were harvested using 5 × Lamelli buffer (25% Glycerol (87% approx.; AnalaR NORMAPUR, cat. 24,385.295), 62.5 mM Tris–HCl buffer (0.5 M, pH 6.8; Biorad, cat. 1,610,799), and 5% SDS (Biorad, cat. 1,610,416) in ddH_2_O), containing 10% DTT (Sigma, cat. 43,816-50ML) and 5% β-mercaptoethanol (Sigma, cat. M3148-25ML) for protein extraction and stored at -20 °C until further processing. Protein content was measured by modified Bradford assay (using detergent compatible Bradford reagent, Thermo Scientific, cat. 1,863,028). Equal amounts (15 µg) of proteins per sample were separated by SDS-PAGE (4–20%; Bio-Rad, cat. 4,568,096) and gels were transblotted onto nitrocellulose membranes (Bio-Rad, cat. 1,620,264). Membranes were blocked with 5% skim milk in TBS for 1 h at room temperature and incubated overnight at 4 °C with anti-Galectin-3 primary antibody (0.5 µg/ml, Invitrogen, cat. 14–5301-85) and anti-vinculin antibody (108 µg/ml, Abcam, cat. 129,002) in 1% skim milk and 0.05% TBS Tween. Afterward, membranes were incubated for 1 h at room temperature with a solution of HRP-conjugated rabbit polyclonal anti-rat antibody (0.2 µg/ml, Invitrogen, cat. 61–9520) in 1% skim milk and 0.05% TBS Tween. Clarity Max Western ECL Substrate (Bio-Rad, cat. 1,705,062) was used to develop Galectin-3 bands on the blots, images were acquired with the ChemiDoc™ Imaging System (Bio-Rad). Further, blots were washed in TBS Tween and incubated for 1 h at room temperature with a solution of HRP-conjugated swine polyclonal anti-rabbit antibody (0.068 mg/ml, Dako, cat. P0399). Vinculin bands together with Galectin-3 bands were developed with Clarity Max Western ECL Substrate. Protein levels of the target bands were calculated by normalizing the optical density of the target on the first developed image calculated by Image Lab 6.1 software (Bio-Rad) to the corresponding optical density of vinculin on the second developed image as loading control. Raw data tables for all executed Western blots can be found in Supplementary data file 1.

### Statistics

Statistical analysis was performed in R using R Studio. For the analysis of GFAP, IBA1, and Galectin-3 immunofluorescence signal, colocalization per retina in both human and rat eyes and cell death analysis of MIO-M1 cells after TNF-a or rotenone treatments, a Shapiro Wilk test was used to test the normality of the data. One-way ANOVA followed by Dunnett’s multiple comparison post hoc test was applied to analyze normally distributed data, Kruskal–Wallis one way analysis of variance followed by pairwise Wilcoxon rank sum test was used to analyze non-normally distributed data. For the comparison of MIO-M1 immunolabeled Galectin-3 signal between primary antibody positive and negative images, and Western blot Galectin-3 optical densities, a Shapiro Wilk test was used to test the normality of the data and subsequently an F-test followed by a Student’s two sample t-test (homoscedastic data) or a Welch’s t-test (heteroscedastic data) was used to compare the mean signal intensities or optical densities of the different groups for normally distributed data, and a Wilcoxon rank-sum test for non-normally distributed data. The P-values and 95% confidence intervals of all comparisons are available in Supplementary Data file 1. For non-parametric tests no 95% confidence intervals were reported. Differences with *P* < 0.05 were considered significant. * = *P* < 0.05, ** = *P* < 0.01, *** = *P* < 0.001. For the box plots, the median is represented by the center hinge with lower and upper hinges indicating the first and third quartiles, whereas whiskers denote 1.5 times the interquartile range.

## Results

### Galectin-3 increases prior to retinal ganglion cell loss in glaucoma and is associated with microglia, astrocytes and Müller glia.

**Fig. 1:**
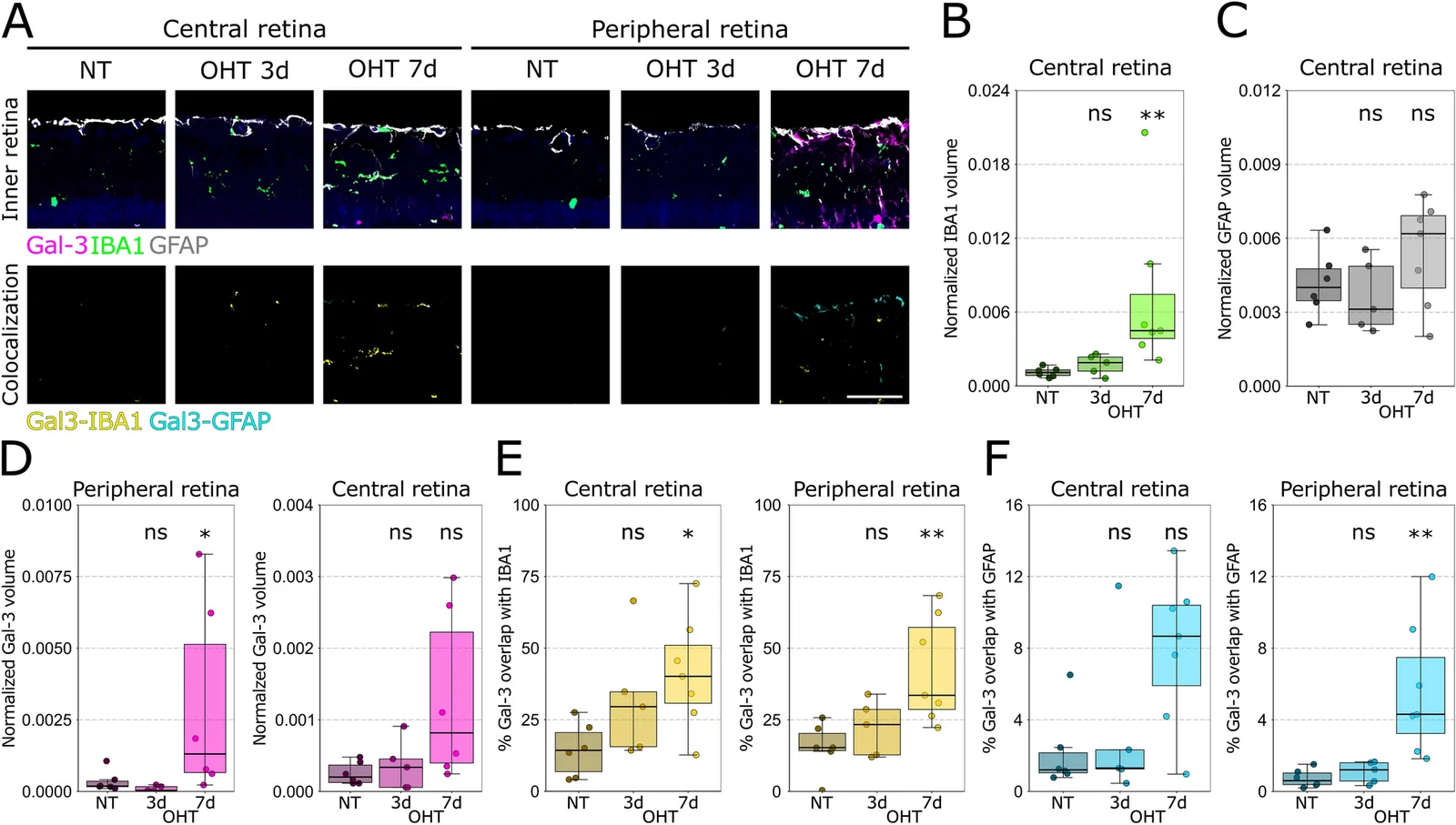
Microglia, astrocytes and Müller glia are associated with Galectin-3 at an early experimental glaucoma stage. **A** Sections were cut from paraffin embedded rat eyes from 3 or 7 days after OHT induction, with normotensive naïve eyes as controls. IBA1, GFAP, and Galectin-3 were immunofluorescently labeled and imaged in the central (± 1000 µm from the optic nerve) and peripheral (± 2500 µm from the optic nerve) retina and volumes of markers and colocalization were calculated. (**B-C**) The volume of IBA1 increased significantly in the central retina at 7d OHT compared to normotensive controls, while the GFAP volume did not. **D** Galectin-3 volume increased significantly in the peripheral retina at 7d OHT compared to normotensive controls. (**E–F**) The colocalization of Galectin-3 to IBA1 increased significantly in the whole retina at 7d OHT compared to normotensive controls, while Galectin-3/GFAP colocalization increased significantly in the peripheral retina at 7d OHT. ns = *P* > 0.05, * = *P* < 0.05, ** = *P* < 0.01, *** = *P* < 0.001. For A, scale bar = 50 µm. Volumes in B-D are normalized to the area of the inner retina per image, and n = retina (where the value is the average of the retina either side of the optic nerve head at the set eccentricity)

Increased Galectin-3 has been identified in multiple animal models of glaucoma at neurodegenerative time points, but earlier time points remain unresolved. We have previously established time points in the rat bead model which represent pre-degenerative OHT (3 days post OHT induction, when IOP is high but there is no detectable retinal ganglion cell degeneration; [46]) and pre- or early- degeneration (7 days post OHT induction, when IOP remains high and molecular changes to retinal ganglion cells are detectable; [44, 47]). However, the neuroinflammatory response at these timepoints had not previously been determined. To address this, we cut new sections from a previously published cohort of rat eyes at these time points [47]. IOP for these eyes is replotted in (Fig S1A). Galectin-3 and glial markers IBA1 (microglia) and GFAP (astrocytes and Müller glia) were labelled and imaged (Fig. 1A, Fig S1B. We detected no change in gross content of either IBA1 or GFAP at 3d OHT compared to NT controls in central (*P* = 0.329 and *P* = 0.834 respectively; Fig. 1B-C) or peripheral retina (*P* = 0.9868 and *P* = 0.108 respectively; Fig S1C-D), suggesting that significant gliosis has yet to occur at this pre-degenerative time point. At 7d OHT, IBA1 content was significantly increased in both central (*P* = 0.0035; Fig. 1B) and peripheral retina (*P* = 0.0111; Fig S1C), but GFAP was not (*P* = 0.372 and *P* = 0.751 respectively; Fig. 1C, Fig S1D). This suggests an early increase in microglial volume, consistent with microglial proliferation and a shift towards pro-inflammatory responses. Galectin-3 labelling demonstrated a similar temporal response with a significant increase in volume at 7d OHT in the peripheral retina (*P* = 0.039; Fig. 1D) and with a non-significant but clear shift in distribution towards higher content in central retina (*P* = 0.11; Fig. 1D). Galectin-3 volume remained unaltered at 3d OHT in both central and peripheral retina (*P* = 0.65 and *P* = 0.12 respectively; Fig. 1D). This suggests that Galectin-3 increase coincides with gross inflammation and gliosis, particularly of microglia. However, we observed colocalization of the Galectin-3 signal with both IBA1 and GFAP. Quantification of signal colocalization demonstrated that Galectin-3 predominantly localized to IBA1 over GFAP in NT retina (mean 15.00% and 1.46% respectively; Fig. 1E-F). At 7d OHT colocalization to IBA1 significantly increased in central (*P* = 0.0263; Fig. 1E) and peripheral retina (*P* = 0.00561; Fig. 1E). Colocalization to GFAP at 7d OHT demonstrated a non-significant but clear shift in distribution towards higher content in central retina (*P* = 0.10; Fig. 1F) and a significant increase in the periphery (*P* = 0.00389; Fig. 1F). These data suggest neuroinflammation at a pre- or early degenerative timepoint in the rat bead model of glaucoma, characterized by microglial proliferation and hypertrophy. Additionally, these data support that Galectin-3 increases at a pre- or early degenerative time point, and that while this coincides with increased microglial content, it is not restricted to microglia and may represent a pan-glial response to OHT in the retina.

### Galectin-3 is associated with Müller glia in human glaucoma

**Fig. 2:**
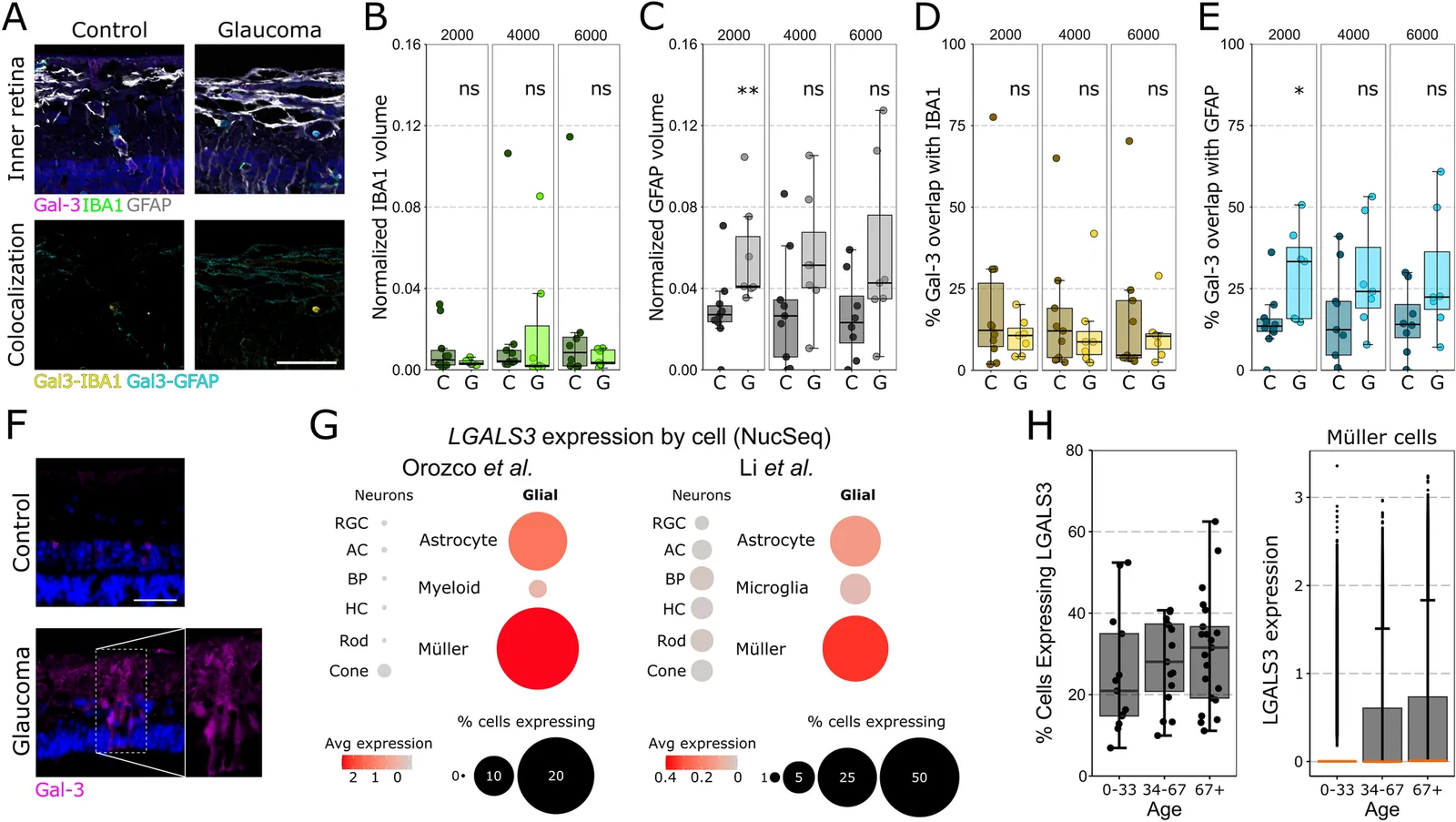
Galectin-3 association with astrocytes and Müller glia is greater in human glaucoma. **A** Retinal paraffin sections of a highly preserved cohort of human donor eyes were immunofluorescently labeled for IBA1, GFAP, and Galectin-3 and imaged at ± 2000 (central retina; shown here), ± 4000 (mid-peripheral retina; Fig S2A), and ± 6000 µm (peripheral retina; Fig S2A) from the optic nerve and volumes of markers and colocalization were calculated. (**B-C**) The volume of IBA1 did not increase significantly at any eccentricity in the retina of glaucoma eyes compared to controls, while the GFAP volume did in the central retina. (**D-E**) The Galectin-3/IBA1 colocalization did not increase significantly at any eccentricity in the retina of glaucoma eyes compared to controls, while the Galectin-3/GFAP colocalization did in the central retina. **F** Immunofluorescent labeling of Galectin-3 in human glaucomatous tissue reflects Müller glia morphology. **G** Single nucleus sequencing demonstrates that Müller glia have the highest average expression and highest percentage of cells expressing *LGALS3* in the normal human retina in two independent datasets. **H** The percentage of Müller glia expressing *LGALS3* by individual retina does not change with age, but *LGALS3* expression per individual Müller glia is increased in older individuals. ns = *P* > 0.05, * = *P* < 0.05, ** = *P* < 0.01, *** = *P* < 0.001. For A and F, scale bar = 50 µm. Volumes in B-C are normalized to the area of the inner retina per image and n = retina (where the value is the average of the retina either side of the optic nerve head at the set eccentricity in glaucoma, and only the side opposite the tumour in controls)

Next, we sought to determine whether this relationship was similar in human glaucoma. Increased Galectin-3 protein levels have been identified in the human glaucomatous optic nerve head [1] and in glaucoma patient aqueous humour [24], but its expression in glaucomatous human retina has yet to be fully explored. We used a well preserved and characterized cohort of human donor eyes in which we previously identified significantly increased inflammation in glaucoma cases [30], Galectin-3, IBA1 and GFAP were labelled (Fig. 2A, Fig S2A. In these eyes, IBA1 content was not significantly changed in central, mid-peripheral, or peripheral retina compared to controls (*P* = 0.67, *P* = 0.30, and *P* = 0.46 respectively; Fig. 2B), whereas GFAP content was significantly increased in the central retina (*P* = 0.0031; Fig. 2C). Galectin-3 content was not significantly changed at any eccentricity (*P* = 0.997, *P* = 0.528, and *P* = 0.825 respectively; Fig S2B), but its colocalization demonstrated a large shift from IBA1 in controls towards GFAP in glaucoma. Galectin-3 colocalization with IBA1 was not significantly altered across all eccentricities (*P* = 0.67, *P* = 0.54, and *P* = 0.92 respectively; Fig. 2D) whereas colocalization to GFAP was significantly increased in the central retina (*P* = 0.0207; Fig. 2E). Galectin-3 positive cell soma were clearly visible in the inner nuclear layer in controls, while in glaucoma retina, whole Müller glia morphology from the outer nuclear layer border to the inner limiting membrane was clearly visible through Galectin-3 labelling alone (Fig. 2F). Supporting this, examination of two publicly available nuclear RNA sequencing datasets from normal human retina [12, 21]) demonstrated that Müller glia have the highest average expression and highest percentage of cells expressing *LGALS3* in the retina (followed by astrocytes, then microglia) (Fig. 2G). In the Human Retina Cell Atlas dataset, donors were subdivided by age (0–33, 34–67, >67 ) and while the percentage of Müller glia expressing *LGALS3* did not increase with age, the expression within individual Müller glia was higher in older age groups (Fig. 2H). Together, these suggest that in addition to its potential importance to microglia, Galectin-3 is particularly associated with Müller glia in human glaucoma.

To assess whether Galectin-3 expression elevation is a consequence of retinal ganglion cell degeneration in human degenerating retina, we maintained postmortem donor human retina punches ex vivo as explants (n = 2 donors, 21.5 and 29 h postmortem). The explants were either processed immediately after dissection (D0) or maintained for 7 days in culture (D7). D7 explants demonstrated a significant loss of 42% of retinal ganglion cells compared to D0 (*P* = 0.049, pairwise-comparisons between punches from the same donor retina (Fig. S2C). Western blot analysis demonstrated that Galectin-3 was not elevated at D7 compared to D0 (*P* = 1; Fig. S2D), however this was donor dependant with eyes from donor 1 demonstrating a 135 and 1549% increase and eyes from donor 2 demonstrating an 86 and 88% decrease. These results suggest that Galectin-3 expression may be driven by glaucoma specific insults or individual differences rather than retinal ganglion cell degeneration per se.

### Galectin-3 is expressed, but not secreted in untreated human Müller glia in vitro

**Fig. 3:**
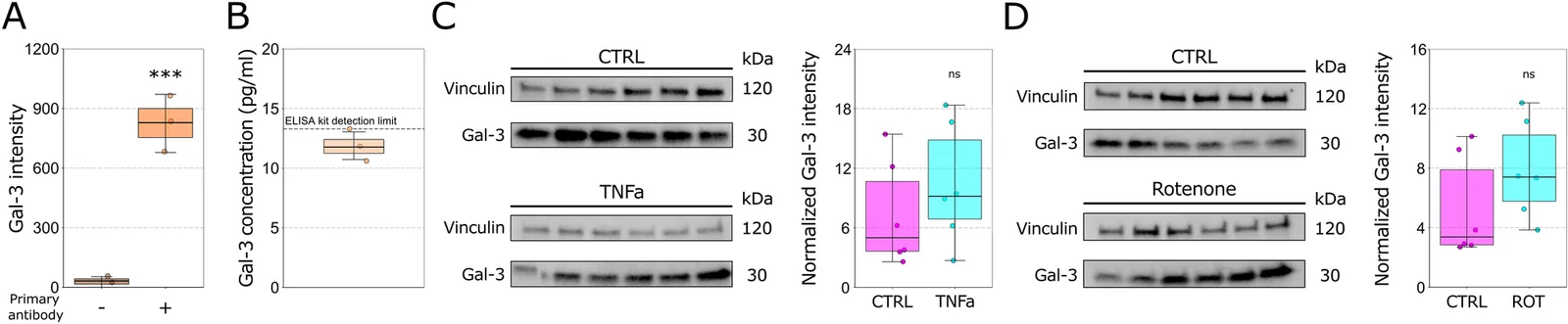
Galectin-3 is expressed, but not secreted in MIO-M1 Müller glia*,* and is unchanged in response to TNFα or sub-lethal rotenone. **A** Immunofluorescent staining of Galectin-3 in untreated human MIO-M1 Müller glia identified significantly higher Galectin-3 intensity in primary antibody positive compared to primary antibody negative cells. **B** ELISA resulted in Galectin-3 concentrations under the detection limit in MIO-M1 medium 72 h after seeding. MIO-M1 Müller glia exposed to **C** TNFα (50 ng/mL) for 24 h or** D** rotenone (0.1 µM) for 24 h demonstrated no change in Galectin-3 protein expression supporting that neither glaucoma-relevant pro-inflammatory signalling or sub-lethal metabolic dysfunction induce Galectin-3 expression in vitro in Müller glia

To confirm the association of Galectin-3 and human Müller glia, we used the immortalized human Müller glia line, MIO-M1. Galectin-3 expression was confirmed by immunofluorescent labeling where Galectin-3 primary antibody positive cells demonstrated a significantly higher signal intensity compared to primary antibody negative cells (P < 0.001; Fig. 3A; Fig S3A). Galectin-3 has a wide array of intracellular and extracellular functions [14]. To determine if Galectin-3 is secreted by human Müller glia, we collected cell medium of MIO-M1 cells 72 h after seeding and executed a Galectin-3 ELISA. The Galectin-3 concentration in the cell medium was under the detection limit of the ELISA kit, suggesting that MIO-M1 Müller glia do not secrete Galectin-3 under basal conditions (Fig. 3B). These data support that the Galectin-3 colocalization to GFAP detected in human central retina is likely to be of Müller glia origin.

To assess whether Müller glia upregulate Galectin-3 in response to specific glaucoma-relevant stimuli, we exposed MIO-M1 cells to a sublethal concentration of either TNF-α (50 ng/ml; a pro-inflammatory protein which is increased in animal models of glaucoma, ocular fluids in glaucoma patients, and post-mortem tissue from glaucoma patients [43] or rotenone (0.1 µM; an inhibitor of Complex I which models reduced oxidative phosphorylation, metabolic dysfunction and stress, and Complex I deficiencies in glaucoma patients [43]) for 24 h (no cell death was detected at these concentrations, Fig S3B. Western blot quantification of Galectin-3 protein levels demonstrated no significant difference in Galectin-3 levels following either TNF-α or rotenone exposure (*P* = 0.368 and *P* = 0.128 respectively; Fig. 3C-D, Fig S3C-D). This suggests that upregulation of Galectin-3 in MIO-M1 Müller glia does not occur in response to these specific pro-inflammatory or metabolism compromising stimuli, but does not preclude changes in response to other or broader pro-inflammatory or metabolic stimuli.

### Galectin-3 expression is regulated in response to phagocytosis in Müller glia

**Fig. 4:**
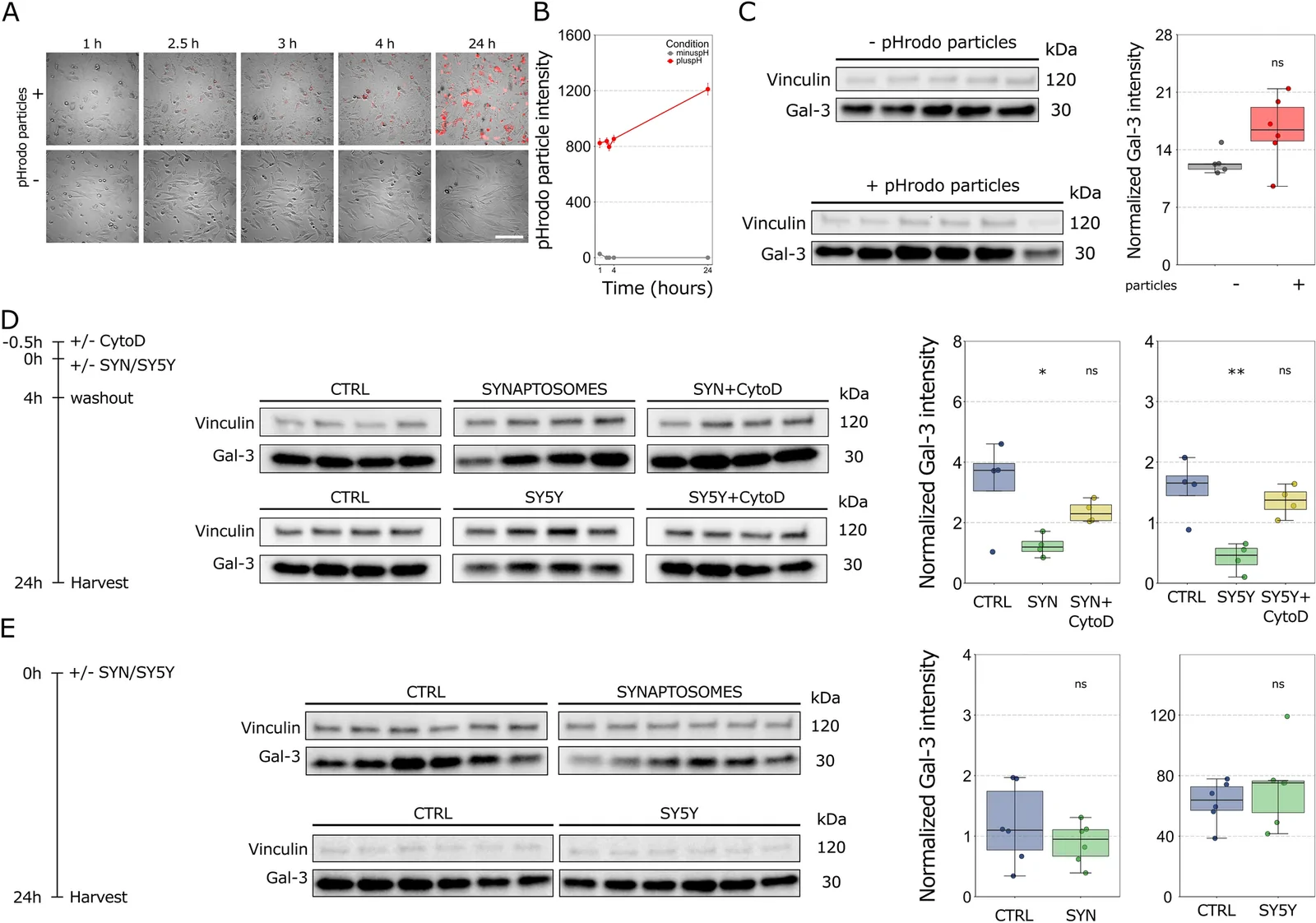
Galectin-3 expression is altered based on phagocytic state and context in MIO-M1 Müller glia. (**A-B**) The treatment of MIO-M1 cells with pHrodo phagocytic particles demonstrated a significant time-dependent increase of pHrodo signal compared to pHrodo particle untreated cells, confirming phagocytosis can occur rapidly and is maintained when excess material is present. **C** Galectin-3 Western blot of MIO-M1 cell lysate following 24 h of exposure did not demonstrated a significant change in Galectin-3 protein levels. **D** Exposure to either mouse cortical synaptosomes or apoptotic neuronal fragments (rotenone exposed SY5Ys) for 4 h followed by a washout significantly lowered Galectin-3 expression, but this was blocked by Cytochalasin D. **E** This did not occur in MIO-M1s exposed continually for 24 h, supporting a mechanism whereby successful phagocytic resolution results in Galectin-3 expression changes. ns = *P* > 0.05, * = *P* < 0.05, ** = *P* < 0.01, *** = *P* < 0.001. For D, scale bar = 200 µm. Galectin-3 intensity is Galectin-3 optical band density normalized to the optical band density of vinculin per lane. For all analyses, n = a single well

Given that Galectin-3 is recognized as a phagocytic marker in both peripheral immune cells [31], neurodegenerative contexts [3], and astrocytes in the optic nerve [19], we hypothesized that Galectin-3 upregulation in Müller glia in human glaucoma retina may be linked to increased Müller glia phagocytosis. To mechanistically probe this, we assessed Galectin-3 expression after inducing phagocytosis in MIO-M1 Müller glia by exposure to inactivated *E. coli* particles conjugated with the fluorophore pHrodo™ Red, a pH-sensitive indicator which only becomes fluorescent in a low pH environment (i.e. when the particle is internalized, and localized in a cellular endosome or lysosome). A time series of MIO-M1 cells treated with phagocytic particles demonstrates a significant time-dependent increase of pHrodo signal confirming increased phagocytosis (*P* < 0.001; Fig. 4A-B), supporting that MIO-M1s were capable of acting as non-professional phagocytes as expected, and could internalize material in lysosomes or endosomes within an hour, continuing for 24 h with continual exposure. Intracellular Galectin-3 protein, quantified by Western blot 24 h after exposure, was not significantly increased in phagocytic particle exposed Müller glia compared to the untreated control group (*P* = 0.07035; Fig. 3C; Fig S4A).

To assess this in a neurodegenerative relevant context, we purified mouse cortex synaptosomes or apoptotic neuronal fragments (differentiated human SH-SY5Ys exposed to Rotenone for 24 h). MIO-M1 Müller glia exposed to these for 4 h (with washout), demonstrated a significant downregulation of Galectin-3, 24 h after initial exposure to synaptosomes (*P* = 0.0243) and apoptotic neuronal fragments (*P* = 0.00238) (Fig. 4D, Fig S4B-C). This change was blocked by co-incubation with Cytochalasin D (a reversible actin cytoskeleton disruptor which blocks phagocytosis, *P* = 0.3312 and P = 0.62227 relative to control for synaptosomes and apoptotic neuronal fragments respectively; Fig. 4D, Fig S4B-C) demonstrating a phagocytosis dependent mechanism. There were no significant changes in Galectin-3 expression following 24 h exposure (without washout) to synaptosomes (*P* = 0.350, Fig. 4E, Fig S4D-E) or apoptotic neuronal fragments (*P* = 0.4266, Fig. 4E, Fig S4D-E). We could not include Cytochalasin D controls here due to toxicity from prolonged exposure. These results suggest that Galectin-3 expression in Müller glia is dynamically altered in response to phagocytic states in a context dependent manner.

Collectively, these data suggest that Galectin-3 upregulation occurs in astrocytes and Müller glia in addition to microglia, particularly in human glaucoma. We demonstrate that Müller glia do not upregulate Galectin-3 expression in response to specific glaucoma relevant stimuli in vitro, but Galectin-3 expression is dynamically altered based on phagocytic sates and context in vitro, supporting the need for further investigation to understand its upregulation in vivo.

## Discussion

Increased Galectin-3 has previously been identified at degenerative stages of experimental [15, 45] and human [1, 24] glaucoma. Galectin-3 might play an important role in glaucoma pathology, since the pharmacological or genetic inhibition in these rodent models results in neuroprotection [15, 27]. However, the mechanisms underlying this remain unresolved. We demonstrate an association between Galectin-3 and microglia, astrocytes, and Müller glia in the retina at a pre- or early degenerative disease stage in a rat model of glaucoma, and a pronounced Galectin-3 association with Müller glia in late-stage human glaucoma. We demonstrate that Galectin-3 upregulation does not occur in response to glaucoma-relevant stimuli in vitro in human Müller glia, but is dynamically altered by phagocytic state.

We previously identified increased Galectin-3 by protein array at the 14 day OHT degenerative timepoint in the rat [9, 45]. Whether it emerges earlier in the disease and inflammatory process was not known. Colocalization of IBA1 and Galectin-3 increased significantly in the whole retina at day 7 OHT, coinciding with the significant increase in retinal IBA1 volume, suggesting Galectin-3 production by reactive microglia at a pre- to early degenerative stage in experimental glaucoma. This is consistent with findings from Margeta et al*.*, who demonstrated *Lgals3* upregulation as a marker of neurodegenerative microglia (MGnD) in 1-year-old DBA/2 J mice [15], a time point typically characterized by moderate to severe retinal ganglion cell degeneration, and co-labelling of Galectin-3 and P2ry12 + cells in bead injected mice. Our labelling demonstrates that Galectin-3 upregulation emerges earlier within microglia and may precede MGnD phenotypes. However, we cannot definitively confirm this since Galectin-3 is known to be highly expressed in monocytes and monocyte-derived macrophages which infiltrate the central nervous system [48], which we cannot differentiate from microglia with IBA1. The increase in Galectin-3 volume at day 7 OHT coinciding with microglial hypertrophy, rather than an earlier timepoint preceding microglial morphology changes, suggests that Galectin-3 acts as a neuroinflammatory aggravator and marker rather than as the initial driver. Consistent with this, when we treated OHT rats with the Galectin-3 inhibitor TD139 (delivered at 3d OHT) we detected no significant changes to microglial morphology at 14 days [27], supporting that microglial morphological reactivity is independent of Galectin-3. TD139 did provide significant retinal ganglion cell neuroprotection, suggesting that Galectin-3 inhibition may prevent other downstream inflammatory insults. Collectively, these data suggest the upregulation of Galectin-3 in early inflammation preceding retinal ganglion cell degeneration in experimental glaucoma, particularly related to microglia.

As well as the association of Galectin-3 with microglia, a significant increase of Galectin-3 co-localization to GFAP was detected in the rat at day 7 after OHT induction, suggesting the production of Galectin-3 by astrocytes and Müller glia. Increased Galectin-3/GFAP colocalization in the retina of human glaucoma eyes compared to controls, more pronounced than in rat OHT eyes, and in the absence of increased IBA1 volume or Galectin-3/IBA1 colocalization as seen in the rat support the production of Galectin-3 by astrocytes and Müller glia in human glaucoma. Emergence of Galectin-3 in astrocytes and Müller glia, at a pre- or early- degenerative timepoint in the rat, and at least in late disease in humans, expands the potential scope of the role of Galectin-3 in glaucoma pathophysiology. Astrocytic upregulation of Galectin-3 in response to significant injury and neurodegeneration in the mouse optic nerve in glaucoma [19] and multiple sclerosis [5], and in reactive proliferating astrocytes in injured mouse cerebral cortex [34] has previously been reported. Additionally, Galectin-3 upregulation by Müller glia was detected at a pre-degenerative timepoint in a mouse model of retinal photoreceptor degeneration [11], supporting that these responses are likely early and lasting. According to Perero et al*.*, Müller glia from peripheral retina have a higher inflammatory reactivity compared to those in the central retina [23], which may explain the increase in Gal-3 and Galectin-3/GFAP colocalization that we observed in the rat peripheral retina as opposed to the centralretina. In the human, the much greater association of Galectin-3 with astrocytes and Müller glia may represent a shift in Galectin-3 responses in later stages of disease, since all human glaucoma retina tested have significant retinal ganglion cell loss and optic nerve head excavation, and amaurosis. However, the detection of Galectin-3 in what are likely astrocyte processes in the nerve fiber layer and Müller glia bodies in the inner nuclear layer in control retina, supports that Galectin-3 has normal physiological roles within these cells, consistent with high *LGALS3* expression in these cells (to a greater extent than microglia) in normal human retina. This enrichment for Galectin-3 in astrocytes and Müller glia in humans (particularly in glaucoma) compared to rats may reflect species differences, age differences, or disease state and stage differences. Additionally, these data might reflect a greater importance of microglial Galectin-3 in early glaucoma pathogenesis, while later stage Müller glia expression of Galectin-3 may be a consequence of retinal degeneration or prolonged pro-inflammatory states. By sub-setting donors from the Human Retina Cell Atlas sequencing dataset by age, we determined that expression of *LGALS3* was higher in Müller glia from old donors. Further experiments comparing Galectin-3 protein levels in eyes enucleated from young and old individuals of the same species would be needed to fully characterize the cause of this difference. We cannot exclude that staining of IBA1 is potentially underreported due to less pervasive staining than when previously using immunohistochemistry in the same eyes [30], which may also underrepresent Galectin-3 colocalization to IBA1. Collectively, these data support an association between Galectin-3 and astrocytes and Müller glia in glaucoma, that is much more pronounced in human than in rat.

In some instances, complete Müller glia morphology was visible by only Galectin-3 immunolabeling in human glaucomatous sections, while cell bodies in the inner nuclear layer were clearly labelled in control retina. This, combined with snRNA sequencing data demonstrating that Müller glia have the highest basal expression of Galectin-3, supports that this Galectin-3/GFAP colocalization is highly likely to mark Galectin-3 production by Müller glia themselves, rather than binding or internalization of microglial derived Galectin-3. Confirming this, the immortalized human Müller glia line MIO-M1 expresses Galectin-3 (as assessed by immunolabeling and Western blot). However, this may not reflect true in vivo expression by Müller glia since MIO-M1s may have altered gene expression from repeated passage (all under P30) or because of the influence of their progenitor like state. Confirmation of this in primary human Müller glia would further strengthen this, but may also be caveated by post-mortem effects and donor comorbidities. The use of rat primary Müller cells would aid in understanding the species differences observed in our histology, but would require adult cultures to avoid developmental influence. Galectin-3 is expressed in various cell types and has many roles, including non-pathogenic/homeostatic functions e.g. cell growth [6, 37] and endocytosis [16, 33], which explains why a basal level of Galectin-3 is present in human Müller glia at a transcript and protein level in donor tissue and MIO-M1s.

Galectin-3 is known as a phagocytic marker in both peripheral immune cells [31], optic nerve astrocytes [4, 19], and in neurodegenerative contexts [3]. We demonstrated higher Galectin-3 protein levels in cells which phagocytosed E.coli particles compared to unexposed cells, linking Müller glia Galectin-3 expression to phagocytosing states. This finding aligns with previous studies, demonstrating Galectin-3 upregulation associated with phagocytosis in not only professional phagocytes such as microglia [5], but also non-professional phagocytes such as astrocytes in the optic nerve [4, 19]. While exogenous Galectin-3 has been demonstrated to promote clearance phagocytosis of photoreceptor outer segments via MERTK in primary murine Müller glia and MIO-M1 cells [11], we demonstrate that phagocytosis itself promotes Galectin-3 expression changes intracellularly. Galectin-3 can act intracellularly on the K-Ras-dependent PI3K signaling pathway to coordinate the cytoskeletal rearrangement necessary for engulfment and phagocytosis [29]. Supporting a phagocytosis dependent mechanism, Cytochalasin D (a reversible actin cytoskeleton disruptor) blocked Galectin-3 expression change following exposure to synaptosomes or apoptotic neuronal fragments. Unexpectedly, Galectin-3 expression was downregulated following short-term exposure, but not following longer-term exposure (24 h). This difference may have arisen from the washout and subsequent 20 h in normal media before Galectin-3 was quantified, which may therefore represent differing responses to phagocytic resolution. Many pro-inflammatory actors (e.g. IL1β, IL8, IL10, GM-CSF and TNFα) are known to be downregulated following efficient phagocytic clearance of apoptotic cells, reflecting a mechanism that supports the resolution of inflammation [50]. Galectin-3 expression may be regulated in a similar fashion upon successful phagocytic clearance, but this may fail with chronic exposure. Establishing this will require addition of further cues that are present in the neurodegenerative milieu of the retina. In glaucoma, phagocytic clearing of dead cells or defective mitochondria [35] is vital for retinal ganglion cell survival. Supporting the nuance of this context, a decrease of phagocytic capacity has been identified in optic nerve head microglia in early experimental glaucoma [42], molecular signaling from microglia to Müller glia has been demonstrated to impact Müller glia autophagy in glaucoma [51], and Müller glia have been demonstrated to upregulate phagocytosis when microglia are absent or insufficient in zebrafish [40]. The role of Galectin-3 in glaucoma remains to be further elucidated, particularly in human glaucoma, and this will be necessary before advancing strategies to target Galectin-3 towards clinical use.

## Conclusion

In conclusion, we demonstrate the upregulation of Galectin-3 in microglia occurs at an early pre-degenerative time point in experimental glaucoma, and this is accompanied by increased Galectin-3 in Müller glia. This increase in Müller glia Galectin-3 is more pronounced in late human glaucoma. Galectin-3 expression is dynamically linked to Müller glia phagocytic states, although this may differ in vivo. These findings provide further insight into inflammatory responses in glaucoma pathology, and their potential roles in retinal ganglion cell dysfunction, degeneration, and neuroprotection.

## Supplementary Information

Supplementary materials

Supplementary data file 1

## Data Availability

All data generated during this study are included in this published article and its supplementary information files. The nuRNAseq dataset from Orozco *et al.*, analyzed in the current study is available in the Gene Expression Omnibus (GEO) repository, through NCBI GEO ([https://www.ncbi.nlm.nih.gov/geo/query/acc.cgi?acc=GSE135133]). Reference number: GSE135133. The nuRNAseq dataset from Li *et al.*, analyzed in the current study is available in the Human Cell Atlas Data Portal repository (Human Retina Cell Atlas v1.0: available at https://data.humancellatlas.org/hca-bio-networks/eye/atlases/retina-v1-0).

## Abbreviations

TD139: 3,3-Deoxy-3,3-bis-(4-[m-fluorophenyl]-1H1,2,3-triazol-1-yl)-thio-digalactoside
BSA: Bovine serum albumin
ELISA: Enzyme-linked immunosorbent assay
FBS: Fetal bovine serum
GFAP: Glial fibrillary acidic protein
IOP: Intraocular pressure
IBA1: Ionized calcium-binding adaptor molecule 1
MGnD: Neurodegenerative microglia
NT: Normotensive
OHT: Ocular hypertension
PBS: Phosphate-buffered saline
