# Supplementary material for: Galectin-3 is elevated in Müller glia in human glaucomatous eyes and ocular hypertensive rat eyes and associated with phagocytosing states
Source: Acta Neuropathol Commun. 2026 Aug 4;14:159. doi: 10.1186/s40478-026-02388-7 (PMC13439881; doi:10.1186/s40478-026-02388-7)
Supplement: Supplementary file 1 [file 40478_2026_2388_MOESM1_ESM.pdf]

Supplementary materials

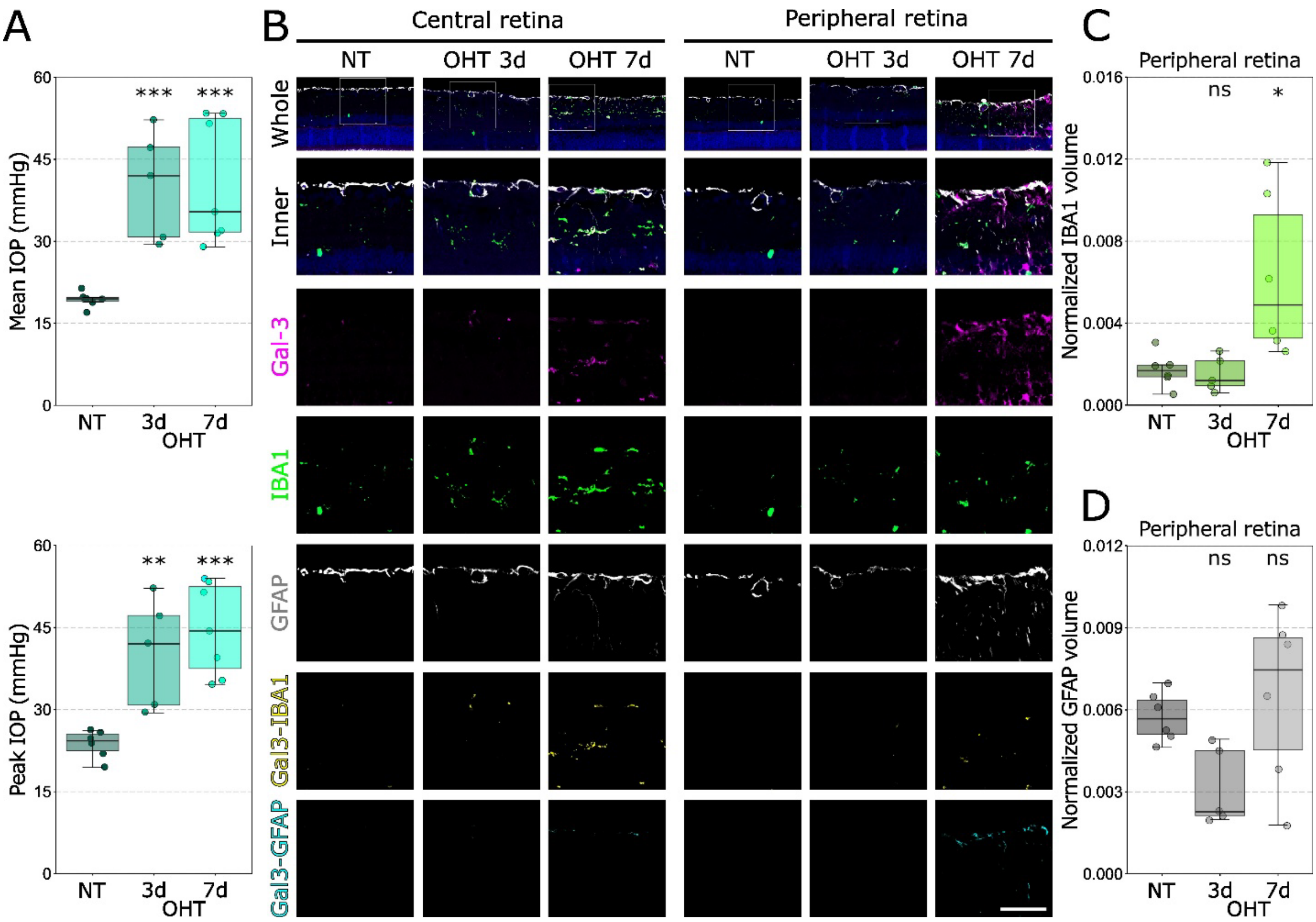

Figure S1. Microglia, astrocytes and Müller cells are associated with Galectin-3 at an early experimental glaucoma stage. A) IOP of rats reached a peak 3 days after OHT induction, and remained high until 7 days after OHT induction compared to normotensive control eyes. **B**) IBA1, GFAP and Galectin-3 were labeled and imaged in the central (±1000 μm from the optic nerve) and peripheral (±2500 μm from the optic nerve) retina, all channels are shown. **C-D**) The volume of IBA1 increased significantly in the peripheral retina at 7d OHT compared to normotensive controls, while the GFAP volume did not. ns = P > 0.05, * = P < 0.05, ** = P < 0.01, *** = P < 0.001. For B, scale bar = 50 μm. Volumes in C-D are normalized to the area of the inner retina per image.

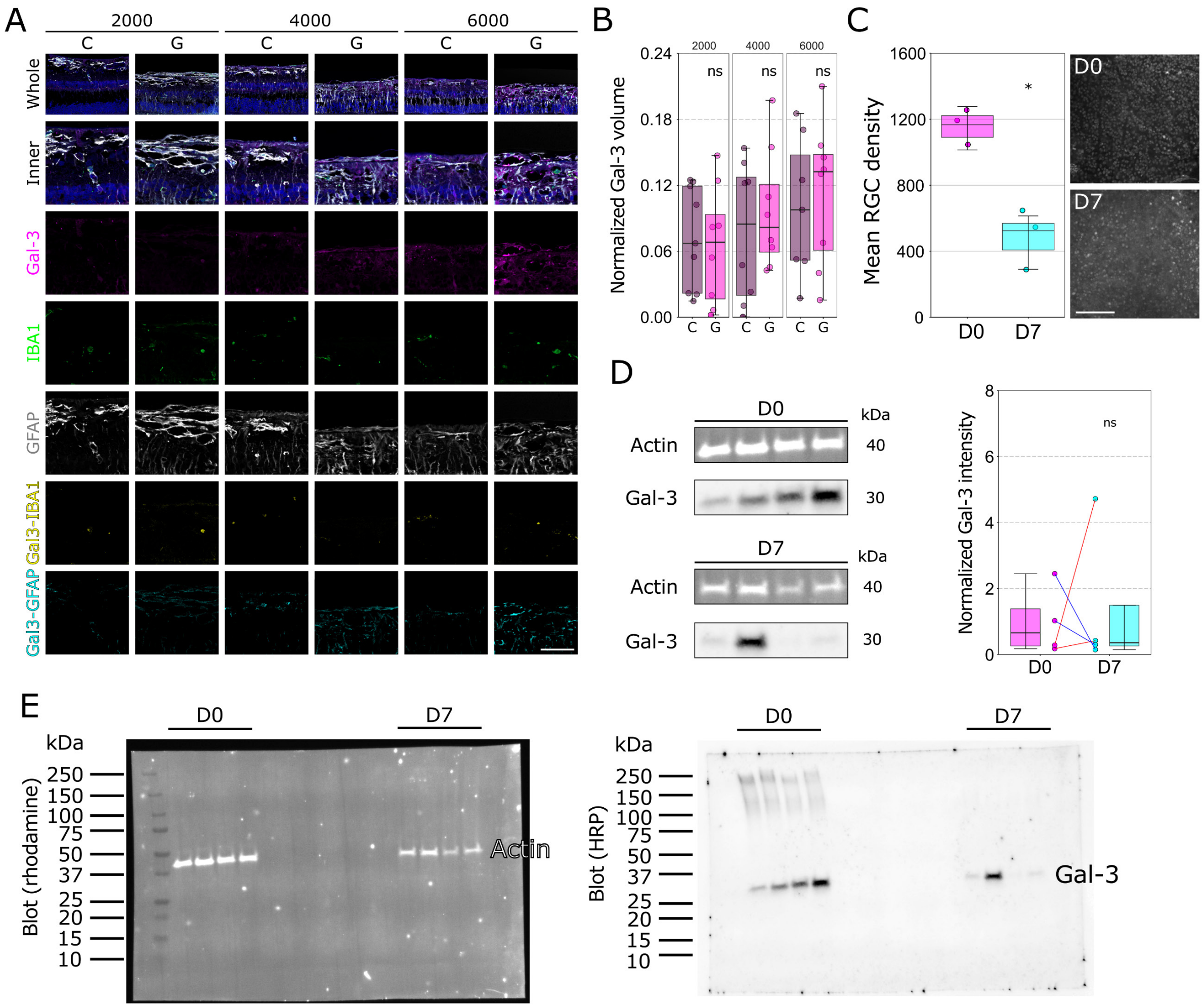

Figure S2. Galectin-3 associates more with astrocytes and Müller cells in human glaucoma. A) Retinal paraffin sections of a highly preserved cohort of human donor eyes were labeled for IBA1, GFAP, and Galectin-3 and imaged at ±2000 (central retina), ±4000 (mid-peripheral retina), and ±6000 μm (peripheral retina) from the optic nerve. **B**) The volume of Galectin-3 did not significantly change at any eccentricity in glaucoma eyes compared to control eyes. **C**) Punches of donor human retina were collected at (21.5-29h postmortem) (D0) or maintained ex vivo for 7 days (D7) at which point RGC density was significantly reduced. **D**) This was not accompanied by a significant change in Galectin-3, although retinas from one donor demonstrated a clear increase (red) and the other a clear decrease (blue). **E**). Original uncropped images of Galectin-3 Western blots and Actin control demonstrating Galectin-3 protein levels

(a distinct immunoreactive band at ±30 kDa, the expected molecular weight of Galectin-3) in whole retinal lysate of D0 and D7 retina punches. ns = $P > 0.05$, * = $P < 0.05$, ** = $P < 0.01$, *** = $P < 0.001$. Scale bar = 50 μm for A; 200 μm for C. Volumes in B are normalized to the area of the inner retina per image.

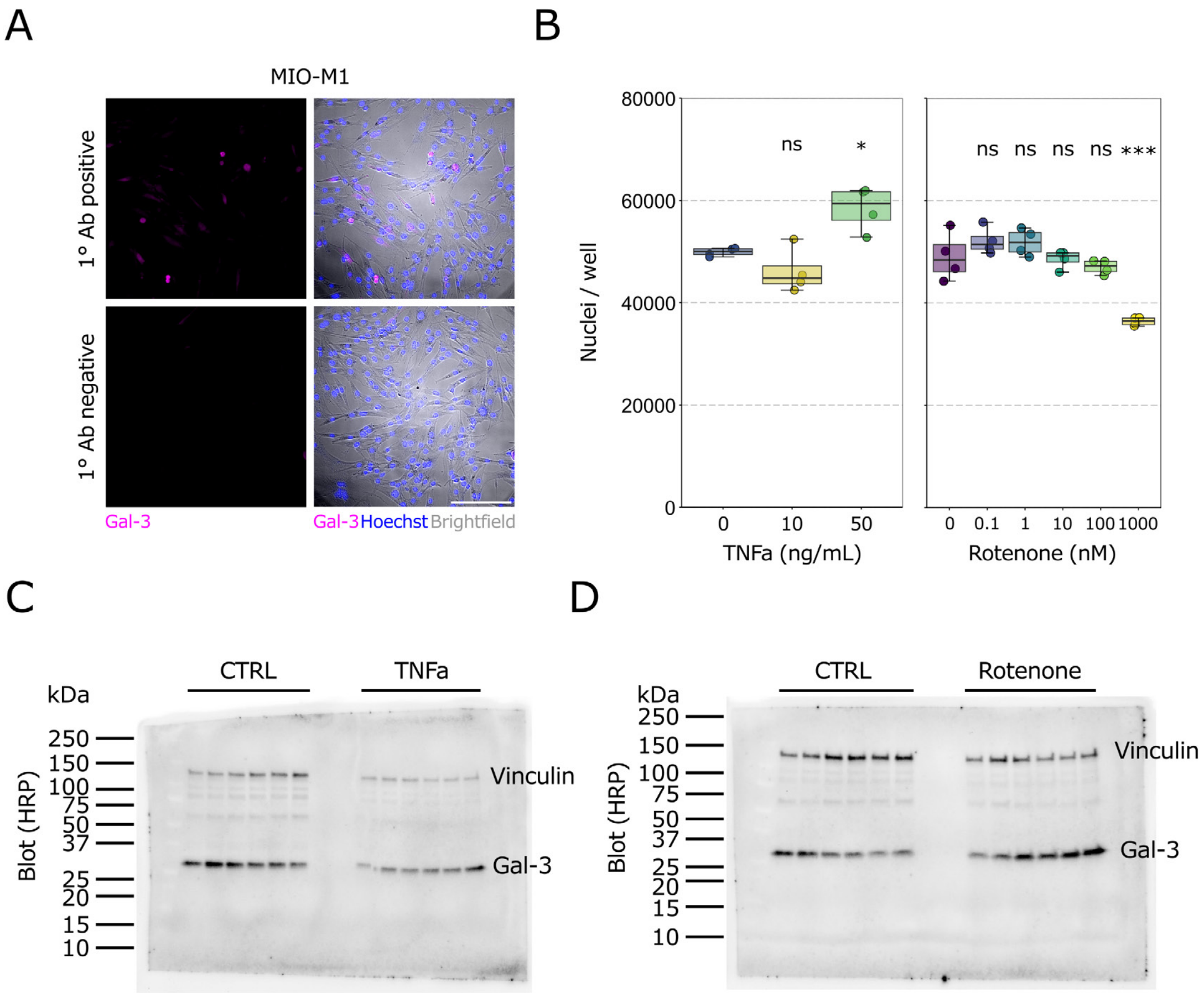

Figure S3. Galectin-3 is expressed, but not secreted in MIO-M1 Müller cells, and but is not altered under glaucoma-relevant stimuli. A) Galectin-3 was labeled in untreated human MIO-M1 Müller cells with primary antibody negative cells as control. **B**) MIO-M1 cells were exposed to TNfa or rotenone and cell survival assessed by particle analysis of DAPI labelled nuclei. TNFa at 10 ng/mL had no effect on cell survival, while 50 ng/mL resulted in significant cell increases, reflecting increased proliferation. Rotenone caused significant cell death only at 1μM. **C-D**) Original images of Galectin-3 Western blot for TNFa and Rotenone treated MIO-M1 Müller cells. ns = P > 0.05, * = P < 0.05, *** = P < 0.001. For A, scale bar = 200 μm.

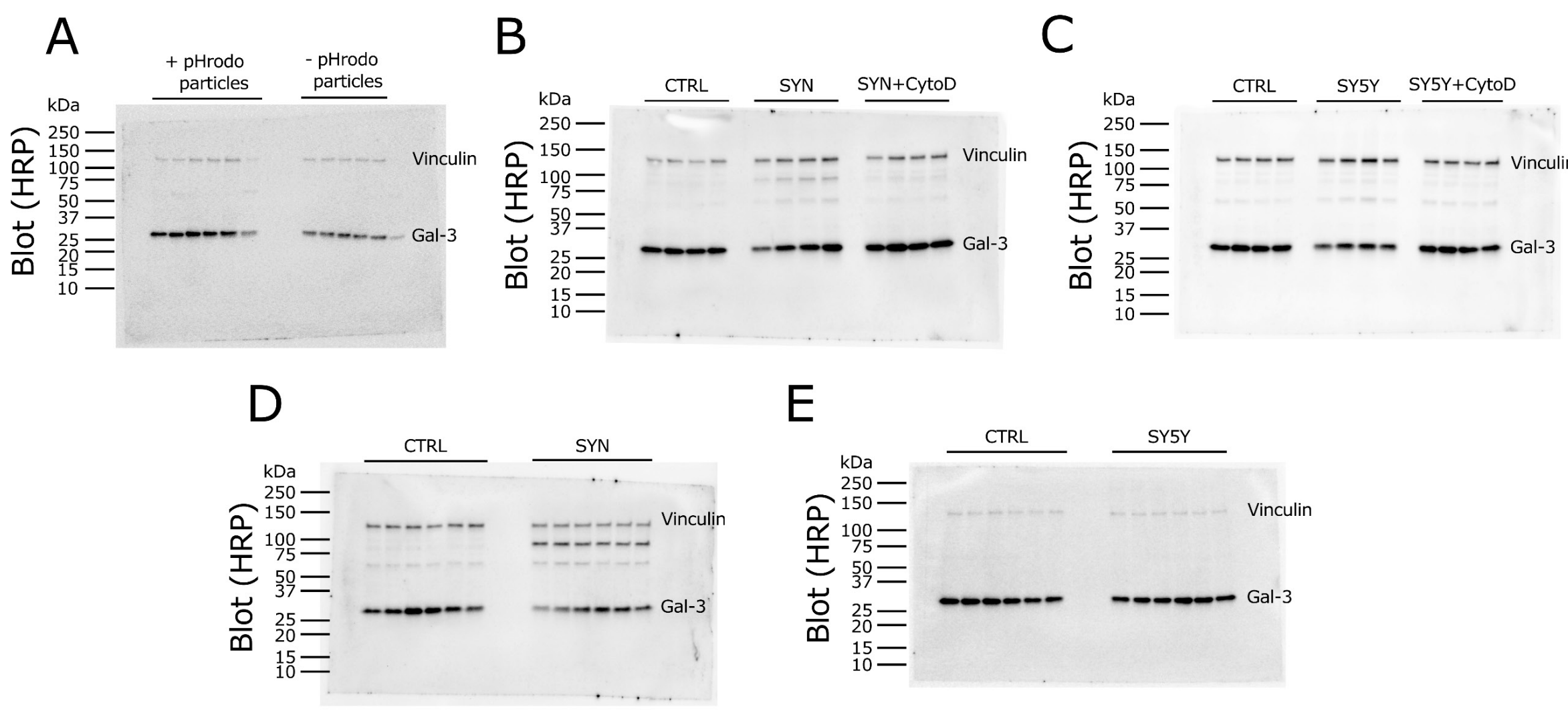

Figure S4. Galectin-3 expression changes following phagocytosis in MIO-M1 Müller cells. Original uncropped images of Galectin-3 Western blots demonstrating Galectin-3 protein levels (a distinct immunoreactive band at ±30 kDa, the expected molecular weight of Galectin-3) in cell lysate of **A**) pHrodo phagocytic particle treated MIO-M1 cells compared to untreated cells, **B**) mouse cortex synaptosomes +/- Cytochalasin D treated MIO-M1 cells compared to untreated cells, **C**) apoptotic fragments of differentiated SY5Ys +/- Cytochalasin D treated MIO-M1 cells compared to untreated cells, **D**) mouse cortex synaptosome treated MIO-M1 cells compared to untreated cells, **C**) apoptotic fragments of differentiated SY5Y treated MIO-M1 cells compared to untreated cells.
